# Risk of Bias in Systematic Reviews of Non-Randomized Studies of Adverse Cardiovascular Effects of Thiazolidinediones and Cyclooxygenase-2 Inhibitors: Application of a New Cochrane Risk of Bias Tool

**DOI:** 10.1371/journal.pmed.1001987

**Published:** 2016-04-05

**Authors:** Anja Bilandzic, Tiffany Fitzpatrick, Laura Rosella, David Henry

**Affiliations:** 1 Dalla Lana School of Public Health, University of Toronto, Toronto, Ontario, Canada; 2 Ontario Strategy for Patient-Oriented Research Support Unit, Toronto, Ontario, Canada; 3 Public Health Ontario, Toronto, Ontario, Canada; 4 Institute for Clinical Evaluative Sciences, Toronto, Ontario, Canada; 5 Institute of Health Policy, Management and Evaluation, University of Toronto, Toronto, Ontario, Canada; Western Sydney University, AUSTRALIA

## Abstract

**Background:**

Systematic reviews of the effects of healthcare interventions frequently include non-randomized studies. These are subject to confounding and a range of other biases that are seldom considered in detail when synthesizing and interpreting the results. Our aims were to assess the reliability and usability of a new Cochrane risk of bias (RoB) tool for non-randomized studies of interventions and to determine whether restricting analysis to studies with low or moderate RoB made a material difference to the results of the reviews.

**Methods and Findings:**

We selected two systematic reviews of population-based, controlled non-randomized studies of the relationship between the use of thiazolidinediones (TZDs) and cyclooxygenase-2 (COX-2) inhibitors and major cardiovascular events. Two epidemiologists applied the Cochrane RoB tool and made assessments across the seven specified domains of bias for each of 37 component studies. Inter-rater agreement was measured using the weighted Kappa statistic. We grouped studies according to overall RoB and performed statistical pooling for (a) all studies and (b) only studies with low or moderate RoB. Kappa scores across the seven bias domains ranged from 0.50 to 1.0. In the COX-2 inhibitor review, two studies had low overall RoB, 14 had moderate RoB, and five had serious RoB. In the TZD review, six studies had low RoB, four had moderate RoB, four had serious RoB, and two had critical RoB. The pooled odds ratios for myocardial infarction, heart failure, and death for rosiglitazone versus pioglitazone remained significantly elevated when analyses were confined to studies with low or moderate RoB. However, the estimate for myocardial infarction declined from 1.14 (95% CI 1.07–1.24) to 1.06 (95% CI 0.99–1.13) when analysis was confined to studies with low RoB. Estimates of pooled relative risks of cardiovascular events with COX-2 inhibitors compared with no nonsteroidal anti-inflammatory drug changed little when analyses were confined to studies with low or moderate RoB. The exception was a rise in the relative risk associated with ibuprofen from 1.07 (95% CI 0.97–1.18) to 1.14 (95% CI 1.03–1.26). The main limitation of our study was testing the instrument on a narrow range of pharmacoepidemiological studies; we cannot assume our findings extend to a broader range of interventions and settings.

**Conclusions:**

The Cochrane RoB tool highlighted a wide range of risks of bias in studies included in two widely cited reviews and had the potential to change the conclusions of the reviews. Systematic reviews that incorporate non-randomized studies of medical interventions should include a detailed assessment of RoB for each included study.

## Abstract 2

David Henry and colleagues re-evaluate findings from two systematic reviews using the new ACROBAT-NRSI bias assessment tool for non-randomized studies.

## Editors' Summary

**Background:**

In the past, clinicians used their own experience to help them make decisions about the best treatments (interventions) for their patients. Nowadays, “evidence-based medicine”—largely based on findings from randomized controlled trials (RCTs)—guides most clinical decisions. RCTs—studies that compare outcomes in groups of patients chosen at random to receive different interventions—are the best way to assess the efficacy of an intervention (the performance of a treatment under ideal conditions), but individual trials often fail to show a statistically significant difference (a difference unlikely to have arisen by chance) between two interventions. Significant differences between interventions can be detected, however, by undertaking a systematic review (a study that identifies all the RCTs on a given intervention using predefined criteria) and a meta-analysis (a statistical technique for combining, or “synthesizing,” the findings from several independent RCTs).

**Why Was This Study Done?:**

Systematic reviews of healthcare interventions can also include non-randomized studies, which use administrative databases to identify people receiving different interventions and electronic health records to determine clinical outcomes. However, non-randomized studies of interventions are prone to many “biases” that affect the accuracy of their findings. For example, a potential bias in non-randomized studies is “confounding,” the possibility that an unmeasured characteristic shared by the people receiving a specific intervention, rather than the intervention itself, is responsible for the observed outcome. When undertaking systematic reviews and meta-analyses, it is essential to measure the risk of bias (RoB) in each individual study included in the review and meta-analysis. But, although a widely used tool is available for measuring RoB in RCTs, bias is seldom considered in detail when synthesizing the results of non-randomized studies of interventions. Here, the researchers assess the reliability and usability of ACROBAT-NRSI, a tool developed by Cochrane (an organization that promotes evidence-informed health decision-making) for the assessment of RoB in non-randomized intervention studies. ACROBAT-NRSI assists authors in identifying potential concerns across seven bias domains and assesses the overall RoB of individual non-randomized intervention studies.

**What Did the Researchers Do and Find?:**

Two of the researchers independently applied the ACROBAT-NRSI process to 37 papers included in two widely cited systematic reviews of non-randomized studies of the relationship between the use of thiazolidinediones (drugs used to treat diabetes, such as rosiglitazone and pioglitazone) and cyclooxygenase-2 (COX-2) inhibitors (nonsteroidal anti-inflammatory drugs [NSAIDs] such as ibuprofen) and major cardiovascular events (heart attack [myocardial infarction] and heart failure). The two researchers largely agreed on their RoB assessments (good inter-rater agreement), which, after training and early experience, took roughly 2.5 hours to complete for each study. In the thiazolidinedione review, six studies had low overall RoB, four had moderate RoB, four had serious RoB, and two had critical RoB. In the COX-2 inhibitor review, two studies low overall RoB, fourteen had moderate RoB, and five had serious RoB. When the researchers restricted meta-analysis to studies with low or moderate RoB, estimates of the pooled relative risks of cardiovascular events with COX-2 inhibitors (compared with no NSAID) changed little, except for a rise in the relative risk associated with ibuprofen. Finally, although the risk estimates for myocardial infarction, heart failure, and death for rosiglitazone compared with pioglitazone remained significantly raised when analyses were confined to studies with low or moderate RoB, there was no significantly increased risk of myocardial infarction when the analysis was confined to studies with low RoB.

**What Do These Findings Mean?:**

These findings show that there was considerable variability in RoB among the studies included in two systematic reviews of non-randomized intervention studies. Although all 37 studies included in these reviews were originally considered to be of sufficiently high quality for inclusion using less comprehensive—or less RoB-focused—critical appraisal tools, only eight were judged to have low RoB using ACROBAT-NRSI. Notably, exclusion of studies with moderate, serious, or critical RoB resulted in clinically important changes to some of the conclusions of the original reviews. Because the researchers considered only two systematic reviews, their findings may not be generalizable—ACROBAT-NRSI needs further testing across a range of study types. Moreover, because the tool is designed to be used within a team setting, studies are needed to investigate whether the performance of the tool depends on the team’s skill mix. Importantly, however, these findings highlight the importance of including a detailed RoB assessment for each study included in systematic reviews of non-randomized studies of medical interventions.

**Additional Information:**

This list of resources contains links that can be accessed when viewing the PDF on a device or via the online version of the article at http://dx.doi.org/10.1371/journal.pmed.1001987.

- More information about ACROBAT-NRSI (A Cochrane Risk of Bias Assessment Tool for Non-Randomized Studies of Interventions) is available; the main Cochrane website provides information about Cochrane and its work; the *Cochrane Handbook for Systematic Reviews of Interventions* has a chapter on including non-randomized studies in systematic reviews
- Wikipedia has pages on evidence-based medicine, clinical trials, systematic review, and meta-analysis (note that Wikipedia is a free online encyclopedia that anyone can edit; available in several languages)
- ClinicalTrials.gov, the US National Institutes of Health clinical trials registry, provides additional background information about clinical trials

## Introduction

Well-conducted randomized controlled trials (RCTs) remain the gold standard for assessing medical interventions because their design controls both measured and unmeasured confounding variables. Systematic reviews with meta-analyses of RCTs have become the accepted evidence base for many important clinical and policy decisions. The limitations of RCTs are well documented [1–3]. They may not reflect “real world” patient experiences because they study highly selected populations in atypical settings. Also, despite substantial investments of time and money, few trials enroll the number of patients over the necessary length of time to quantify uncommon or long-term outcomes.

Non-randomized studies of interventions have proliferated in recent years due to increased access to extensive linked administrative databases and electronic health records, with large populations, long follow-up periods, and advances in analytic approaches to control for confounding [4,5]. It is recognized that non-randomized studies provide different information (i.e., “real world” effectiveness, wider population inclusion, and longer follow-up) from RCTs [3]. Thus, the methods can be considered complementary, and systematic reviews of both types of studies are needed to provide a comprehensive assessment of a body of evidence.

However, controversy persists. While there is agreement that large, high-quality non-randomized studies can accurately quantify adverse outcomes of medical treatments [6], there is less agreement on their capacity to generate unbiased estimates of the effectiveness of medical interventions [7]. Nevertheless, non-randomized studies are increasingly being included in systematic reviews and meta-analyses [8]. The large sample sizes of many non-randomized studies correspond to greater weight attributed to their findings during statistical pooling. The concern is that, while the larger sample sizes may increase precision in summary estimates of treatment effects, they may also be prone to bias [8]. In order to minimize this problem, it is necessary to measure the risk of bias (RoB) in the individual studies that are being included in systematic reviews. This enables exclusion of studies that have an increased RoB from the overall estimate, or during sensitivity analyses.

While a widely used gold-standard RoB tool exists for RCTs [9], there is less agreement on how to assess RoB within non-randomized study designs. A wide variety of checklists, judgment ratings, and scales for observational studies have been proposed [10,11], including the Newcastle–Ottawa Scale (NOS) [12], the Downs and Black checklist [13], and the Scottish Intercollegiate Guidelines Network’s methodology checklists [14]. None of these tools reflects a contemporary domain-based approach to bias assessment, and they are dated (e.g., the current version of the popular NOS was released in 2000) [11,12]. Many instruments use overall rating scales, which have been shown to be flawed [15].

To address this problem, the Cochrane Collaboration released a draft of a comprehensive tool specifically for non-randomized studies in September 2014 [16]. The Cochrane Risk of Bias Tool for Non-Randomized Studies of Interventions (ACROBAT-NRSI) builds upon the Cochrane Risk of Bias tool for RCTs [9] and assesses internal validity through a series of RoB judgments in seven chronologically organized domains to provide an overall RoB assessment for each study (see Box 1).

**Box 1. Domains of Bias Assessed by ACROBAT-NRSI [16]**

**Domains of bias**

Pre-intervention (baseline)

1 Bias due to confounding
2 Bias in selection of participants into the study

At intervention

3 Bias in measurement of interventions

Post-intervention

4 Bias due to departures from intended interventions
5 Bias due to missing data
6 Bias in measurement of outcomes
7 Bias in selection of the reported result

**Judgment about RoB for each domain**

1. Low RoB: the study is comparable to a well-performed randomized trial with regard to this domain
2. Moderate RoB: the study is sound with regard to this domain, but cannot be considered comparable to a well-performed randomized trial
3. Serious RoB: the study has some important problems in this domain
4. Critical RoB: the study is too problematic in this domain to provide any useful evidence on the effects of intervention

Overall RoB judgment for each study*

1. Low RoB: the study is judged to be at low RoB for all domains
2. Moderate RoB: the study is judged to be at low or moderate RoB for all domains, and moderate in at least one domain
3. Serious RoB: the study is judged to be at serious RoB in at least one domain, but not at critical RoB in any domain
4. Critical RoB: the study is judged to be at critical RoB in at least one domain

*Reviewers have some discretion in making an overall risk of bias judgment based on the assessment of individual domains. To quote from the ACROBAT guidance document: “In practice some ‘Serious’ risks of bias (or ‘Moderate’ risks of bias) might be considered to be additive, so that ‘Serious’ risks of bias in multiple domains can lead to an overall judgement of ‘Critical’ risk of bias (and, similarly, ‘Moderate’ risks of bias in multiple domains can lead to an overall judgement of ‘Serious’ risk of bias).”

The first aim of this study was to assess the performance of ACROBAT-NRSI by applying it to the studies included in two published systematic reviews of the adverse cardiovascular effects of thiazolidinediones (TZDs) [17] and cyclooxygenase-2 (COX-2) inhibitors [18]. The second aim was to determine whether limiting the meta-analyses to studies with lower RoB changed the overall estimates of the adverse drug effects.

## Methods

### ACROBAT-NRSI

The ACROBAT-NRSI instrument considers each non-randomized study as an attempt to emulate a hypothetical randomized trial (the “target trial”) that compares the health effects of two or more interventions. The ACROBAT-NRSI guidance points out that the target trial need not be feasible or ethical, and recommends that it is useful to consider the population, interventions, comparators, and outcomes of such a hypothetical trial [16]. It is also important to decide whether the target trial would be analyzed according to initial treatment assignment (“intention to treat” analog) or according to both initiation and adherence to treatment (“per protocol” analog).

Users of the instrument are guided through seven chronologically arranged (pre-intervention, at intervention, and post-intervention) bias domains (See Box 1). Signaling questions help flag potential bias concerns and help review authors make RoB judgments. The first three domains (pre-intervention and at intervention) are specific to non-randomized studies of interventions, whereas the remaining four domains also have relevance to the assessment of RoB in RCTs (Box 1). Signaling questions for the bias domains are framed so that “yes” indicates a lower RoB than “no” (e.g., “Did the authors use an appropriate analysis method that adjusted for all the critically important confounding domains?”). If the answers to all signaling questions for a domain are “yes” or “probably yes,” then the overall RoB is judged to be low. The ACROBAT-NRSI instrument is provided as S1 Table.

### Selection of Systematic Reviews and Meta-analyses

We selected two widely cited systematic reviews with meta-analyses that addressed important questions about the safety of widely used prescription drugs: one by Loke, Kwok and Singh [17], who investigated the cardiovascular risks of TZDs (comparing rosiglitazone to pioglitazone) in diabetic patients, and one by McGettigan and Henry [18], who investigated the cardiovascular risks associated with a range of selective and nonselective COX-2 inhibitors, with non-use of the drug class as the reference.

We considered reviews of drug effects an appropriate subject for initial testing as they are generally simple interventions that do not involve complexities such as operator skill or extensive infrastructure. Cardiovascular outcomes are clear-cut and not prone to major misclassification. We chose reviews with a substantial number of component studies to give us a sample size sufficient to assess inter-rater agreement. In both reviews. the majority of studies used patient data from large, population-based administrative health databases, and most used sophisticated methods to adjust results for bias. We knew that the quality scores for the component studies in the COX-2 inhibitor review (using the popular NOS) were tightly grouped, with high overall scores [18,19]. Further, the results of both reviews were broadly similar to those from meta-analyses of randomized trials [17,18]. We considered that they would provide a good test of the responsiveness of the new tool to modest variations in RoB and allow us to assess consequential effects of bias on the pooled estimates of adverse effects associated with use of these drugs.

We retrieved full-text copies of the 39 component studies included in the two reviews. One study of TZDs (Graham et al. [20]), was used in a training and calibration exercise that involved all four authors. This article was chosen as it had been assessed during ACROBAT-NRSI working group meetings and majority consensus ratings had been established. All four authors applied ACROBAT-NRSI (version 1.0.0) to this study and met to compare and discuss judgments, interpretations of the guidance document, and user experiences.

Two reviewers (A. B. and T. F.) independently assessed the remaining component studies. Both authors are trained epidemiologists, but had no prior experience using ACROBAT-NRSI. For reports with multiple risk estimates (for a single outcome), the results cited in the original systematic review were extracted and their corresponding properties were assessed (combination of variables in the statistical model, exposure definition, etc.). Two study reports in the form of abstracts [21,22] were excluded, as they did not contain enough information to be assessed by the instrument. This left 37 articles to be evaluated. Individual study assessments were conducted independently, and results recorded, before a meeting at which reviewers compared judgments and achieved a consensus. If both raters had the same category of judgment for a domain, no further discussion occurred. If the ratings differed, each rater provided their reasoning for selecting their RoB judgment. The supporting notes (written in the comments area of the tool) were useful for recalling details relevant to RoB judgment. Inter-rater reliability was measured for each domain of bias, and for the overall RoB judgment, by calculating weighted Kappa scores using linear weighting in SAS 9.4 [23].

The meta-analyses from each review were replicated using RevMan 5.3 before and after RoB assessment. In the case of McGettigan and Henry [18], the risk estimates from the two studies available only in abstract form were excluded from both the before and after RoB assessment analyses. All three cardiovascular outcomes from Loke et al. [17] were assessed, as well as each individual nonsteroidal anti-inflammatory drug (NSAID) group exposure in relation to the major cardiovascular outcome in McGettigan and Henry [18].

Generic inverse-variance weighting was used in a random effects model, as set out in the methods sections of the original study reports. This exercise included all eligible studies. Next, those studies judged as having an overall serious or critical RoB were excluded from the meta-analyses, leaving only estimates from studies with overall low or moderate RoB. In a further analysis, moderate RoB studies were also excluded, resulting in meta-analyses of only low RoB studies. The heterogeneity in the meta-analyses was measured using the *I* ^2^ statistic, and changes in this statistic, the risk estimates, and their confidence intervals were recorded between the original meta-analysis and the re-analyses stratified by RoB. We made an informal assessment of usability by asking reviewers to record the time taken to complete evaluations and to record their overall impressions of using the ACROBAT-NRSI instrument.

## Results

Details of the 37 studies included in the two reviews are provided in Tables 1 and 2. Seventeen studies were analyzed as cohorts and 20 as case–control designs; the majority of the latter were nested in cohorts. In total, 34/37 (92%) studies were performed using linked administrative claims data or electronic medical records. Risk estimates varied across studies and outcomes. However, the majority of estimates were 1.00 or greater. In the case of TZDs, 28/31 relative risk estimates lay between 1.00 and 1.70. In the case of COX-2 inhibitors, 40/66 relative risk estimates lay between 1.00 and 2.29.

**Table 1:** Details of component studies included in the systematic review by Loke et al. [17].

Study, Year	Country	Study Design	Data Type	Number of Participants	Risk Estimate^†^ (95% CI)			
					Risk Measure	Myocardial Infarction	Heart Failure	Overall Mortality
Bilik et al. [24], 2010	US	Cohort	Admin/MR	*R* = 564, *P* = 334	HR	1.30 (0.31–5.37)	0.69 (0.28–1.69)	—
Brownstein et al. [25], 2010	US	Cohort	EMR	*R* = 1,879, *P* = 806	RR	1.70 (1.10–2.63)	—	—
Dormuth et al. [26], 2009	Canada	Case–control	Admin	Cases = 2,244, controls = 8,903	HR	1.00 (0.67–1.49)	—	—
Graham et al. [20], 2010	US	Cohort	Admin	*R* = 67,593, *P* = 159,978	HR	1.06 (0.96–1.18)	1.25 (1.16–1.34)	1.14 (1.05–1.24)
Hsiao et al. [27], 2009	Taiwan	Cohort	Admin	*R* = 49,624, *P* = 12,010	HR	1.36 (1.22–1.53)*	1.40 (1.15–1.71)*	-
Juurlink et al. [28], 2009	Canada	Cohort	Admin	*R* = 16,951, *P* = 22,785	HR	1.05 (0.90–1.23)	1.30 (1.15–1.45)	1.16 (1.02–1.33)
Koro et al. [29], 2008	US	Case–control	Admin	Cases = 9,870, controls = 29,610	OR	1.12 (0.99–1.26)	-	-
Lipscombe et al. [30], 2007	Canada	Case–control	Admin	Cases = 3,695, controls = 18,351 (myocardial infarction); cases = 3,478, controls = 18,045 (heart failure); cases = 5,529, controls = 18,835 (mortality)	OR	1.27 (1.02–1.58)*	1.38 (1.13–1.69)*	1.13 (0.92–1.38)*
Margolis et al. [31], 2008	UK	Cohort	EMR	*R* = 7,282, *P* = 2,244	HR	1.00 (0.80–1.30)	—	—
Pantalone et al. [32], 2009	US	Cohort	EMR	*R* = 1,079, *P* = 1,508	HR	—	0.84 (0.52–1.35)	1.23 (0.79–1.92)
Stockl et al. [33], 2009	US	Case–control	Admin	Cases = 1,681, controls = 6,653	OR	1.26 (0.79–2.00)	—	—
Tzoulaki et al. [34], 2009	UK	Cohort	EMR	*R* = 140,082, *P* = 45,807	HR	1.34 (0.86–2.09)	1.04 (0.75–1.44)	1.36 (1.05–1.76)
Walker et al. [35], 2008	US	Cohort	Admin	*R* = 57,000, *P* = 51,000	HR	1.21 (0.95–1.54)	—	—
Wertz et al. [36], 2010	US	Cohort	Admin	*R* = 18,319, *P* = 18,309	HR	0.94 (0.75–1.18)	1.10 (0.94–1.31)	1.02 (0.86–1.21)
Winkelmayer et al. [37], 2008	US	Cohort	Admin	*R* = 14,101, *P* = 14,260	IRR	1.08 (0.93–1.25)	1.13 (1.01–1.26)	1.15 (1.05–1.26)
Ziyadeh et al. [38], 2009	US	Cohort	Admin	*R* = 47,501, *P* = 47,501	HR	1.41 (1.13–1.75)	—	—

^†^Relative risk comparing rosiglitazone and pioglitazone use and accompanying 95% confidence intervals, as replicated to the second decimal using RevMan 5.3.

*Unadjusted estimates.

Admin, administrative data; EMR, electronic medical records; HR, hazard ratio; IRR, incidence rate ratio; MR, medical records; OR, odds ratio; *P*, number of pioglitazone users; *R*, number of rosiglitazone users; RR, rate ratio.

**Table 2:** Details of component studies included in the systematic review by McGettigan and Henry [18].

Study, Year	Setting	Study Design	Data Type	Number of Participants	Risk Measure	Risk Estimate^†^ (95% CI)							
						Celecoxib	Rofecoxib	Meloxicam	Naproxen	Diclofenac	Ibuprofen	Indomethacin	Piroxicam
Bak et al. [39], 2003	Denmark	Case–control	Admin	Cases = 4,765, controls = 40,000	OR	—	—	—	0.70 (0.40–1.22)	1.10 (0.70–1.73)	1.30 (1.00–1.69)	1.40 (0.80–2.45)	0.50 (0.20–1.25)
Curtis et al. [40], 2003	US	Cohort	Admin/MR	3,577 users, 6,673 non-users	HR	—		—	—	—	0.84 (0.70–1.01)	—	—
Fischer et al. [41], 2005	UK	Case–control	EMR	Cases = 8,688, controls = 33,923	OR	—	—	—	0.96 (0.66–1.38)	1.23 (1.00–1.51)	1.16 (0.92–1.46)	1.36 (0.82–2.25)	0.95 (0.53–1.69)
Garcia Rodriquez et al. [42], 2000	UK	Case–control	EMR	Cases = 1,013, controls = 5,000	OR	—	—	—	—	—	—	—	—
Garcia Rodriquez et al. [43], 2004	UK	Case–control	EMR	Cases, = 4,975, controls = 20,000	OR	—	—	0.97 (0.60–1.56)	0.89 (0.64–1.24)	1.18 (0.99–1.40)	1.06 (0.87–1.29)	0.86 (0.56–1.32)	1.25 (0.69–2.25)
Gislason et al. [44], 2006	Denmark	Cohort	Admin	29,362 users, 29,070 non-users	OR	2.06 (1.73–2.45)	2.29 (1.99–2.65)	—	—	2.19 (1.93–2.49)	1.39 (1.27–1.53)	—	—
Graham et al. [45], 2005	US	Case–control	Admin	Cases = 8,134, controls = 31,496	OR	0.84 (0.67–1.04)	1.34 (0.98–1.82)		1.14 (1.00–1.30)	—	1.06 (0.96–1.17)	—	—
Hippisley-Cox and Coupland [46], 2005	UK	Case–control	EMR	Cases = 9,128, controls = 86,349	OR	1.21 (0.96–1.54)	1.32 (1.09–1.61)	—	1.27 (1.01–1.60)	1.55 (1.39–1.72)	1.24 (1.11–1.39)	—	—
Johnsen et al. [47], 2005	Denmark	Case–control	Admin	Cases = 10,280, controls = 102,797	OR	1.25 (0.97–1.62)	1.80 (1.47–2.21)	—	1.50 (0.99–2.29)	—	—	—	—
Kimmel et al. [48], 2004	US	Case–control	Ad hoc	Cases = 1,055, controls = 4,153	OR	—	—	—	0.48 (0.28–0.82)	—	0.52 (0.39–0.69)	—	—
Kimmel et al. [49], 2005	US	Case–control	Ad hoc	Cases = 1,718, controls = 6,800	OR	0.43 (0.23–0.79)	1.16 (0.70–1.93)	—	—	—	—	—	—
Lévesque et al. [50], 2005	Canada	Case–control	Admin	Cases = 2,844, controls = 56,880	RR	0.99 (0.85–1.16)	1.24 (1.05–1.46)	1.06 (0.49–2.30)	1.17 (0.75–1.84)	—	—	—	—
MacDonald and Wei [51], 2003	UK	Cohort	Admin	Cases = 822, controls = 6,285	HR	—	—	—	—	0.80 (0.49–1.31)	1.73 (1.05–2.84)	—	—
Mamdani et al. [52], 2003	Canada	Case–control	Admin	66,964 users, 100,000 non-users	RR	0.90 (0.70–1.16)	1.00 (0.80–1.25)		1.00 (0.60–1.67)				
McGettigan et al. [53], 2006	Australia	Case–control	Ad hoc	Cases = 328, controls = 487	OR	1.11 (0.59–2.11)	0.63 (0.31–1.28)	—	—	—	0.98 (0.53–1.81)	—	—
Ray [54], 2002	US	Cohort	Admin	181,441 users, 181,441 non-users	RR	—	—	—	0.95 (0.82–1.09)	—	1.15 (1.02–1.28)	—	—
Ray [55], 2002	US	Cohort	Admin	151,728 users, 202,916 non-users	RR	0.96 (0.76–1.21)	—	—	0.93 (0.82–1.06)	—	0.91 (0.78–1.06)	—	—
Schlienger et al. [56], 2002	UK	Case–control	EMR	Cases = 3,315, controls = 13,139	OR	—	—	—	0.68 (0.42–1.13)	1.38 (1.08–1.77)	1.17 (0.87–1.58)	1.03 (0.58–1.85)	1.65 (0.78–3.49)
Solomon et al. [57], 2002	US	Case–control	Admin	Cases = 4,452, controls = 17,700	ReR	—	—	—	0.84 (0.72–0.98)	—	1.02 (0.88–1.18)	—	—
Solomon et al. [58], 2004	US	Case–control	Admin	Cases = 10,895, controls = 49,044	OR	0.93 (0.84–1.02)	1.14 (1.00–1.31)	—	—	—	—	—	—
Watson et al. [59], 2002	UK	Case–control	EMR	Cases = 809, controls = 2,285	OR	—	—	—	0.57 (0.31–1.06)	1.68 (1.14–4.29)	0.74 (0.35–1.55)	—	—

^**†**^Relative risk of COX-2 inhibitor compared with no-use or remote exposure; accompanying 95% confidence intervals replicated to the second decimal using RevMan 5.3.

Admin, administrative data; EMR, electronic medical records; HR, hazard ratio; MR, medical records; OR, odds ratio; ReR, relative risk; RR, rate ratio.

### Inter-Rater Agreement on Risk of Bias Judgments

The weighted kappa scores varied across the seven domains of bias assessed by ACROBAT-NRSI (Table 3). In the case of the Loke et al. [17], kappa values ranged from 0.59 (bias due to missing data) to 0.91 (bias in selection of participants). The remaining kappa values were between 0.63 and 0.78, indicating substantial agreement between the two raters [60]. For McGettigan and Henry [18], the kappa scores ranged from 0.45 (bias in selection of reported results) to 1.00 (bias due to missing data). The remaining scores were between 0.50 and 0.91, denoting moderate to substantial agreement. For the overall score, the Kappa statistic showed substantial agreement for both studies (0.72 and 0.91).

**Table 3:** Weighted Kappa scores for inter-rater agreement when assessing the component studies included in two systematic reviews.

Systematic Review	Domain							Overall RoB Judgment
	Bias Due to Confounding	Bias in Selection of Participants	Bias in Measurement of Interventions	Bias Due to Departures from Intended Interventions	Bias Due to Missing Data	Bias in Measurement of Outcomes	Bias in Selection of Reported Results	
Loke et al. [17]	0.72	0.91	0.63	0.67	0.59	1.00	0.78	0.72
McGettigan and Henry [18]*	0.78	0.50	0.71	0.77	1.00	1.00	0.45	0.91

*Graham et al. [20] was excluded from these analyses, as it was used for training purposes.

### Risk of Bias Assessments

The consensus judgments for the domains of bias and overall RoB assessments for studies included in the two systematic reviews are given in Tables 4 and 5. Assessment comments are summarized in S2 and S3 Tables. Loke et al. [17] studied three major outcomes (heart failure, myocardial infarction, and death). As the assessments of the RoB domains did not differ by individual outcome, a single set of domain-specific and overall judgments is provided.

**Table 4:** Consensus ACROBAT-NRSI judgments between two reviewers by domain of bias—component studies from Loke et al. [17].

Component Study	Domain							Overall RoB Judgment
	Bias Due to Confounding	Bias in Selection of Participants	Bias in Measurement of Interventions	Bias Due to Departures from Intended Interventions	Bias Due to Missing Data	Bias in Measurement of Outcomes	Bias in Selection of Reported Results	
**Cohort study design**								
Bilik et al. [24]	Serious	Low	Low	Low	Low	Low	Low	Serious
Brownstein et al. [25]	Moderate	Low	Moderate	Moderate	Low	Low	Low	Serious
Graham et al. [20]	Low	Low	Low	Low	Low	Low	Low	Low
Hsiao et al. [27]	Critical	Serious	Low	Moderate	Low	Low	Low	Critical
Juurlink et al. [28]	Low	Low	Low	Low	Low	Low	Low	Low
Margolis et al. [31]	Moderate	Serious	Moderate	Moderate	Low	Low	Low	Serious
Pantalone et al. [32]	Serious	Serious	Low	Moderate	Serious	Low	Low	Critical
Tzoulaki et al. [34]	Low	Low	Low	Low	Moderate	Low	Low	Moderate
Walker et al. [35]	Low	Low	Low	Low	Low	Low	Low	Low
Wertz et al. [36]	Low	Low	Low	Low	Low	Low	Low	Low
Winkelmayer et al. [37]	Low	Low	Low	Low	Low	Low	Low	Low
Ziyadeh et al. [38]	Moderate	Low	Low	Low	Low	Low	Low	Moderate
**Case–control study design**								
Dormuth et al. [26]	Low	Low	Low	Low	Low	Low	Low	Low
Koro et al. [29]	Moderate	Moderate	Low	Low	Low	Low	Serious	Serious
Lipscombe et al. [30]	Moderate	Low	Low	Low	Low	Low	Low	Moderate
Stockl et al. [33]	Moderate	Low	Low	Low	Low	Low	Low	Moderate

**Table 5:** Consensus ACROBAT-NRSI judgments between two reviewers by domain of bias—component studies from McGettigan and Henry [18].

Component Study	Domain							Overall RoB Judgment
	Bias Due to Confounding	Bias in Selection of Participants	Bias in Measurement of Interventions	Bias Due to Departures from Intended Interventions	Bias Due to Missing Data	Bias in Measurement of Outcomes	Bias in Selection of Reported Results	
**Cohort study design**								
Curtis et al. [40]	Moderate	Moderate	Low	Moderate	Moderate	Low	Low	Serious
Gislason et al. [44]	Low	Low	Low	Low	Low	Low	Low	Low
MacDonald and Wei [51]	Moderate	Low	Low	Low	Low	Low	Low	Moderate
Mamdani et al. [52]	Moderate	Low	Low	Low	Low	Low	Low	Moderate
Ray et al. [54]	Moderate	Low	Low	Low	Low	Low	Low	Moderate
Ray et al. [55]	Moderate	Low	Low	Low	Low	Low	Low	Moderate
**Case–control study design**								
Bak et al. [39]	Serious	Low	Low	Low	Low	Low	Low	Serious
Fischer et al. [41]	Moderate	Low	Low	Low	Moderate	Low	Low	Moderate
Garcia Rodriquez et al. [42]	Moderate	Low	Low	Low	Moderate	Low	Low	Moderate
Garcia Rodriquez et al. [43]	Moderate	Low	Low	Low	Moderate	Low	Low	Moderate
Graham et al. [45]	Low	Low	Low	Low	Low	Low	Low	Low
Hippisley-Cox and Coupland [46]	Moderate	Low	Low	Low	Moderate	Low	Low	Moderate
Johnsen et al. [40]	Moderate	Low	Low	Low	Low	Low	Low	Moderate
Kimmel et al. [48]	Moderate	Moderate	Moderate	Low	Moderate	Low	Low	Serious
Kimmel et al. [49]	Moderate	Moderate	Moderate	Low	Moderate	Low	Low	Serious
Lévesque et al. [50]	Moderate	Low	Low	Low	Low	Low	Low	Moderate
McGettigan et al. [53]	Low	Low	Moderate	Low	Low	Low	Low	Moderate
Schlienger et al. [56]	Moderate	Low	Low	Low	Moderate	Low	Low	Moderate
Solomon et al. [57]	Moderate	Moderate	Low	Moderate	Low	Low	Moderate	Serious
Solomon et al. [58]	Low	Moderate	Low	Low	Low	Low	Low	Moderate
Watson et al. [59]	Low	Low	Low	Low	Moderate	Low	Low	Moderate

The overall judgments for the component studies from Loke et al. [17] were distributed across all four rating categories. Six studies were found to be at low RoB. The RoB assessments for the remaining studies were as follows: four moderate, four serious, and two critical ROB. For the component studies in McGettigan and Henry [18], the overall judgments appeared less variable. Fourteen of 21 studies fell into the moderate RoB category. Only two studies were rated as low RoB, and five were deemed to have serious RoB. None of the studies received a critical RoB rating. For both reviews, the main causes of serious or critical overall RoB assessments were weaknesses in the domains of confounding and selection of participants.

### Changes in Risk Estimates and Conclusions

For rosiglitazone compared with pioglitazone, excluding all component studies judged to be have serious or critical RoB resulted in slightly lower risk estimates for myocardial infarction and heart failure outcomes overall (Table 6). Both risk estimates remained elevated and statistically significant. The estimates for overall mortality did not change for either study type (cohort or case–control), or overall. However, when studies judged as having moderate RoB were also excluded from the meta-analysis, the pooled odds ratio estimate for myocardial infarction for rosiglitazone compared with pioglitazone fell from 1.16 (95% CI 1.07–1.24) to 1.06 (95% CI 0.99–1.13). The other outcomes, heart failure and overall mortality, did not change to a material extent.

**Table 6:** Risk estimates from meta-analyses: comparison of original estimates with post-assessment estimates for the systematic review by Loke et al. [17].

Outcome	Original Effect Estimate (95% CI)				Post-Assessment Effect Estimate (95% CI)			
	*n*	Cohort Studies	Case–Control Studies	Overall	*n*	Cohort Studies	Case–Control Studies	Overall
**Analysis A**								
Myocardial infarction	15	1.16 (1.05–1.28)	1.15 (1.04–1.27)	1.16 (1.07–1.24)	10	1.10 (1.02–1.20)	1.21 (1.01–1.45)	1.12 (1.04–1.20)
Heart failure	8	1.22 (1.15–1.29)	1.39 (1.21–1.60)	1.24 (1.16–1.31)	6	1.21 (1.15–1.27)	No change	1.21 (1.14–1.30)
Overall mortality	8	1.14 (1.09–1.20)	1.13 (0.92–1.39)	1.14 (1.09–1.20)	6	No change	No change	No change
**Analysis B**								
Myocardial infarction	15	1.16 (1.05–1.28)	1.15 (1.04–1.27)	1.16 (1.07–1.24)	6	1.06 (0.99–1.13)	1.00 (0.67–1.49)	1.06 (0.99–1.13)
Heart failure	8	1.22 (1.15–1.29)	1.39 (1.21–1.60)	1.22 (1.14–1.31)	4	1.22 (1.16–1.28)	N/A	1.22 (1.16–1.28)
Overall mortality	8	1.14 (1.09–1.20)	1.13 (0.92–1.39)	1.14 (1.09–1.20)	4	1.13 (1.08–1.20)	N/A	1.13 (1.08–1.20)

Analysis A: studies judged to have serious or critical overall RoB were excluded; analysis B: studies scoring moderate, serious, or critical RoB were excluded; *n*: number of studies included.

N/A, not applicable.

Risk estimates for COX-2 inhibitors tended to increase in re-analyses confined to studies judged to be at low or moderate overall RoB, except for indomethacin and meloxicam, which featured in only two studies (Table 7). Risk estimates for the more selective COX-2 inhibitors (celecoxib, rofecoxib) showed little change, with only one study removed from the meta-analyses. For the nonselective NSAIDs, the risk estimates for naproxen, diclofenac, and piroxicam remained similar to the original estimates. The relative risk estimate for ibuprofen increased from 1.07 (95% CI 0.97–1.18) to 1.14 (95% CI 1.03–1.26), indicating an elevated cardiovascular risk after exclusion of four studies assessed as having serious RoB. Due to the low number of studies deemed to have low RoB, we were unable to perform a sensitivity analysis excluding studies judged as having moderate RoB.

**Table 7:** Risk estimates from meta-analyses: comparison of original estimates with post-assessment estimates for the systematic review by McGettigan and Henry [18].

Intervention	Original Estimate (95% CI)				Post-Assessment (95% CI)			
	*n*	Cohort Studies	Case–Control Studies	Overall	*n*	Cohort Studies	Case–Control Studies	Overall
Celecoxib	10	1.22 (0.69–2.15)	0.98 (0.85–1.13)	1.04 (0.85–1.28)	9	No change	1.01 (0.90–1.14)	1.10 (0.90–1.34)
Rofecoxib	9	1.52 (0.68–3.42)	1.29 (1.10–1.50)	1.32 (1.05–1.65)	8	No change	1.29 (1.09–1.53)	1.33 (1.05–1.69)
Meloxicam	2	N/A	0.99 (0.66–1.49)	0.99 (0.66–1.49)	2	N/A	No change	No change
Naproxen	14	0.94 (0.86–1.03)	0.93 (0.79–1.11)	0.95 (0.85–1.07)	11	No change	1.05 (0.89–1.23)	1.01 (0.91–1.14)
Diclofenac	9	1.36 (0.51–3.65)	1.36 (1.21–1.54)	1.40 (1.15–1.70)	8	No change	1.38 (1.22–1.57)	1.43 (1.17–1.75)
Ibuprofen	15	1.12 (0.90–1.39)	1.04 (0.91–1.18)	1.07 (0.97–1.18)	11	1.20 (0.96–1.49)	1.13 (1.05–1.21)	1.14 (1.03–1.26)
Indomethacin	5	N/A	1.22 (1.04–1.43)	1.22 (1.04–1.43)	4	N/A	1.19 (0.98–1.44)	1.19 (0.98–1.44)
Piroxicam	4	N/A	1.05 (0.69–1.59)	1.05 (0.69–1.59)	3	N/A	1.20 (0.83–1.73)	1.20 (0.83–1.73)
Any/other NSAID	18	1.10 (0.94–1.29)	1.10 (0.96–1.27)	1.10 (0.99–1.23)	14	1.15 (0.97–1.36)	1.18 (1.02–1.36)	1.18 (1.06–1.31)

Studies judged to have serious or critical overall RoB were excluded; *n*: number of studies included.

N/A, not applicable.

### Effects on Heterogeneity of Risk Estimates

In the case of Loke et al. [17], *I* ^2^ statistics for the summary risk estimates for myocardial infarction, heart failure, and death changed little after exclusion of studies with critical or serious RoB (from 48%, 41%, and 0% to 19%, 41%, and 0%, respectively). After further exclusion of studies judged to have moderate RoB, there was reduced heterogeneity among the remaining studies (*I* ^2^ statistics: 0%, 16%, and 0%, respectively). No pattern could be seen with the nine individual NSAID analyses after exclusion of studies with critical or serious RoB.

### Usability of Cochrane ACROBAT-NRSI

Initially, reviewers took an average of 4 h (but up to 8 h in one instance) to complete each component study assessment. By the end of the study, and with increased experience with the instrument, most studies were assessed within 2.5 h. The reviewers found that it took longer to assess cohort studies than case–control studies. In part, this was because of difficulty in evaluating the potential for time-varying confounding, as essential information regarding this domain was commonly not reported. Overall, reviewers agreed that important determinants of success in applying the instrument were training in epidemiology, familiarity with certain adjustment methods (e.g., propensity score matching), and the creation of a comprehensive list of potential confounders and co-interventions before starting the assessment.

## Discussion

We found that a comprehensive assessment revealed variability in the RoB in non-randomized studies that were included in two systematic reviews of adverse cardiovascular events associated with the use of TZDs and COX-2 inhibitors. Of all studies included in the reviews, only eight of 37 studies that were considered of sufficiently high quality to be included in the two published systematic reviews were judged to have low RoB. The exclusion of studies with moderate, serious, or critical RoB resulted in changes to some risk estimates—in particular, rosiglitazone was no longer associated with an increased risk of myocardial infarction, while the reverse was true for ibuprofen and cardiovascular events.

### Clinical Relevance

Although the changes in risk estimates after exclusion of poorer quality studies were small, they may be important in a field where decisions are made on the basis of small relative increases in the risk of serious adverse events. In the case of the NSAID meta-analysis, the most notable change was a rise in the relative risk estimate for ibuprofen (compared with no NSAID use). This was a small change, but the risk may be real, as ibuprofen has been shown to be associated with dose-related increases in the relative risk of cardiovascular events in both randomized and non-randomized studies [19]. In the case of rosiglitazone, the summary relative risk estimate (compared with pioglitazone) for myocardial infarction moved towards the null after exclusion of nine studies assessed as having moderate, serious, or critical RoB. This is not consistent with the most recent meta-analyses of RCTs of rosiglitazone [61]. However, the RCTs compared rosiglitazone with placebo, insulin, biguanides, or sulfonylureas, not with pioglitazone. The RoB-stratified estimates of the risk of myocardial infarction with rosiglitazone compared with pioglitazone should not therefore be assumed to conflict with the trial results.

### Comparison with Other Tools to Assess Risk of Bias

The substantial variation in RoB we found in these published systematic reviews indicates that ACROBAT-NRSI is sensitive to variations in bias across a range of studies that were considered to be of sufficiently high quality to be included in the reviews considered here. In the case of the COX-2 inhibitors, the authors of the published review originally assessed the quality of the component studies by applying the NOS. [18,19] Using this scale, they found that all studies ranked highly (seven or eight out of a possible total of nine points on the scale). In contrast, with application of the domain-based ACROBAT-NRSI instrument, five of the studies were assessed as being at serious RoB, 14 at moderate RoB, and only two at low RoB. This comparison reveals two things. First, the NOS scores were too tightly clustered to enable examination of the impact of bias on the pooled risk estimates. Second, the overall rating scale used in the NOS did not reveal weaknesses in specific domains that generated poor overall assessments of RoB with the ACROBAT-NRSI instrument, which does not generate an overall score. A simple summary score implies equal weighting of domains of bias, and the overall score may disguise serious or critical flaws and fail to document where the flaws are occurring. The new Cochrane tool allows a more transparent judgment. The instrument enables the identification and categorization of the severity of domain-specific flaws that are important in determining the overall assessment of RoB.

There are many published instruments for assessing susceptibility to bias in non-randomized studies. While there is general agreement about the key domains that should be assessed in the case of RCTs, this is not so with non-randomized studies [9,11]. This is because non-randomized studies have considerably more opportunities for variation in design and analysis, in addition to RoB due to the lack of random allocation and blinding. In their review, Sanderson and colleagues identified 86 assessment tools for non-randomized studies, comprising 41 simple checklists, 12 checklists with additional summary judgments, and 33 scales [11]. The authors concluded that around half of the published scales did not describe the development process and had not been tested for reliability or validity. As a result, they were unable to recommend a specific instrument.

A recent review by Katikireddi et al. found that the majority of 59 systematic reviews published between March and May 2012 included some form of critical appraisal of the included studies [62]. The percentage was higher for RCTs (71%) than non-randomized studies (57%), which is ironic given that non-randomized studies are more susceptible to bias. Katikireddi et al. found that review authors used a variety of existing and adapted critical appraisal tools but that fewer than half included domain-level RoB assessments and that there was confusion about how these scores and ratings should be included in the synthesis and interpretation of review findings. This underscores the importance of assessing domain-specific RoB, which allows for a more nuanced understanding of biases within individual studies.

### Experience with ACROBAT-NRSI

ACROBAT-NRSI is demanding to use as it addresses the serious and complex issues of RoB in non-randomized studies of healthcare interventions. It took two reviewers approximately 2.5 h to complete the process for each component study, including reading the paper, applying the tool, and achieving consensus. This was after training and early experience with the tool. Proper application of the instrument requires a substantial time and resource commitment in addition to an in-depth understanding of the sources of bias in non-randomized studies. We believe this commitment, including the use of two raters, is necessary because of the complexity of non-randomized studies, the inevitable discrepancies that emerge between ratings, and the value of the consensus process that follows. In our study, the raters were supported by a methods expert (L. R.) and a clinician (D. H.). We think both roles are a necessary part of teams that are evaluating (or conducting) systematic reviews that include non-randomized intervention studies. This RoB assessment effort is justified as the results of these systematic reviews may form the basis of policy or regulatory decisions.

We are aware that broader feedback from other users of the ACROBAT-NRSI instrument has indicated that rewording of some signaling questions within the domains of bias is desirable, and that process is underway. We anticipate that as more people use the instrument, further changes will be needed to improve its usability. It is important that potential users access the most recent version of the instrument (available at http://www.riskofbias.info). Further developments of the instrument are unlikely to change the domains of bias, or how these are assessed. But changes to signaling questions will help guide interpretation. As such, our experiences in this study are relevant to future users of the instrument.

ACROBAT-NRSI has been used to assess the RoB of non-randomized studies included in several recently published systematic reviews [63–66]. We were unable to find another published study that reported on the inter-rater reliability of the instrument or estimated the effect of restricting reviews to studies with low or moderate RoB.

We are aware of three reports (in abstract form) of inter-rater reliability of the instrument presented at the 2015 Cochrane Colloquium in Vienna, Austria. The topic areas were environmental exposure, housing improvements, and the relationship between benzodiazepine use and mortality [67–69]. All studies found lower levels of inter-rater agreement than we did. The differences may have been due to the nature of the literature we reviewed and the fact that our raters were epidemiologists, had received training in the use of the instrument, and had gone through a calibration exercise that included an author involved in the development of ACROBAT-NRSI. The tool may not be so readily used by less qualified or less trained personnel, but, arguably, they should not be evaluating systematic reviews that include non-randomized studies of healthcare interventions.

The information derived from application of ACROBAT-NRSI can be integrated into tools designed to provide overall ratings of systematic reviews. In the case of ROBIS (a tool for assessing the RoB in systematic reviews), the relevant domain is number 3, concerned with individual study appraisal [70]. ROBIS appraises a number of other steps in the review process that can introduce bias, in addition to flaws in the component studies. Likewise, ACROBAT-NRSI can provide information on RoB that can be integrated into the revised version of the popular AMSTAR systematic review critical appraisal instrument [71].

### Limitations

Our study has several limitations. First, ACROBAT-NRSI has not been subject to a formal test of construct validity. That means we cannot be certain that the instrument truly measures the constructs (in this case domains of bias) that it was designed to measure. However, we note that it underwent an extensive development program involving many methods experts, has considerable face validity, and was developed from a well-established and validated instrument (the Cochrane Risk of Bias tool for RCTs). Second, we limited our assessment to two reviews of relatively sophisticated pharmacoepidemiological studies. We cannot assume our findings extend to a broader range of interventions and settings. The instrument needs further testing across a range of study types. Third, many of the studies in the two reviews under consideration used propensity score or other matching methods, and ACROBAT-NRSI and related findings may function differently in non-randomized studies that use alternative methods such as self-controlled designs or interrupted time series analysis. Finally, ACROBAT-NRSI was designed to be used within a team setting, with methodologists and subject matter experts contributing to study evaluations [16]. Our study involved two reviewers with similar training backgrounds, who had access to content expertise. But it is possible that other skill mixes in the reviewers would lead to different RoB judgments.

### Conclusions

Systematic reviews that include non-randomized studies of medical interventions should encompass a detailed assessment of domain-level RoB for each included study. Even in a sophisticated field such as contemporary pharmacoepidemiology, a sensitive rating tool can detect significant variation in RoB between individual studies. Exclusion of studies deemed to have unacceptably high RoB may impact the findings of pooled estimates of intervention effects, altering both the statistical and clinical significance of the results.

## Supporting Information

S1 Table The Cochrane risk of bias tool for non-randomized studies of interventions. (DOCX)

S2 Table Consensus overall risk of bias ratings by study and corresponding reasons for ranking of Loke et al. [17] component studies. (DOCX)

S3 Table Consensus overall risk of bias ratings by study and corresponding reasons for ranking of McGettigan and Henry [18] component studies. (DOCX)

## Abbreviations

ACROBAT-NRSI: Cochrane Risk of Bias Tool for Non-Randomized Studies of Interventions
COX-2: cyclooxygenase-2
NOS: Newcastle–Ottawa Scale
NSAID: nonsteroidal anti-inflammatory drug
RCT: randomized controlled trial
RoB: risk of bias
TZD: thiazolidinedione
