# Supplementary material for: Risk of Bias in Systematic Reviews of Non-Randomized Studies of Adverse Cardiovascular Effects of Thiazolidinediones and Cyclooxygenase-2 Inhibitors: Application of a New Cochrane Risk of Bias Tool
Source: PLoS Med. 2016 Apr 5;13(4):e1001987. doi: 10.1371/journal.pmed.1001987 (PMC4821619; doi:10.1371/journal.pmed.1001987)
Supplement: S1 Table — (DOCX) [file pmed.1001987.s001.docx]

# The Cochrane ROB tool for non-randomised studies of interventions (version 1.0.0)

Note: users should use the current version of the instrument, available at: [www.riskofbias.info](http://www.riskofbias.info)

**6: At protocol stage**

1. **Specify the research question by defining a generic target randomized trial**

Participants

Experimental intervention

Control intervention

1. **Specify the nature of the target comparison (effect of interest)**

e.g. effect of *initiating* intervention (as in an intention-to-treat analysis), or effect of *initiating and adhering to* intervention (as in a per-protocol analysis)

1. **List the confounding domains relevant to all or most studies**
2. **List the possible co-interventions that could differ between intervention groups and could have an impact on study outcomes**

© 2014 by the authors. All rights reserved. Reproduced with the permission of the authors.

**For each study:**

1. **Specify a target trial specific to the study.**

|  |  |  | Participants |  |
| --- | --- | --- | --- | --- |
| The protocol-specified target |  | *OR* | Experimental intervention |  |
|  |  |  |  |  |
| randomized trial fully applies |  |  |  |  |
|  |  |  |  |  |
|  |  |  | Control intervention |  |

1. **Specify the outcome**

Specify which outcome is being assessed for risk of bias (typically from among those earmarked for the Summary of Findings table). Specify whether this is a proposed benefit or harm of intervention.

1. **Specify the effect of interest**

e.g. effect of *initiating* intervention (as in an intention-to-treat analysis), or effect of *initiating and adhering to* intervention (as in a per-protocol analysis)

1. **Specify the specific result being assessed**

In case of multiple alternative analyses being presented, specify the numeric result (e.g. RR = 1.52 (95% CI 0.83 to 2.77) and/or a reference (e.g. to a table, figure or paragraph) that uniquely defines the result being assessed.

1. **Preliminary consideration of confounders**

a. Within each confounding domain listed in the review protocol, list the relevant variables, if any, measured in this study.

b List additional confounding domains, if any, specific to the setting of this particular study. Within each domain, list the relevant variables, if any, measured in this study.

c List additional domains and corresponding measured variables, if any, that the study authors identified as potential confounders that are not included in the above domains.

***7.5.1 Relationship between confounding domains and potential confounders.***

In the table below, “critically important” confounding domains are those for which, in the context of this study, adjustment is expected to lead to a clinically important change in the estimated effect of the intervention. “Validity” refers to whether the confounding variable or variables fully measure the domain, while “reliability” refers to the precision of the measurement (more measurement error means less reliability).

| Confounding | Is the domain | Measured | Did the authors demonstrate that | Is the domain measured validly | OPTIONAL: Is adjusting for this |  |
| --- | --- | --- | --- | --- | --- | --- |
| Domain | critically | Variable | controlling for this variable was | and reliably by this variable (or | variable (alone) expected to |  |
|  | important?* |  | unnecessary?* | these variables)? | move the effect estimate up or |  |
|  |  |  |  |  | down? ** |  |
|  |  |  |  |  |  |  |
|  | Yes / No |  |  | Yes / No / No information | Up / Down / No information |  |
|  |  |  |  |  |  |  |
|  |  |  |  |  |  |  |
|  |  |  |  |  |  |  |
|  |  |  |  |  |  |  |
|  |  |  |  |  |  |  |

- In the context of a particular study, variables can be demonstrated not to be confounders and so not included in the analysis: (a) if they are not predictive of the outcome; (b) if they are not predictive of intervention; or (c) because adjustment makes no or minimal difference to the estimated effect of the primary parameter. Note that “no statistically significant association” is not the same as “not predictive”.
- For example, if the crude effect estimate is 1.3, adjustment to 1.6 is up, while adjustment to 0.7 is down. If the effect estimate is 0.7, adjustment to 1.1 is up while adjustment to 0.4 is down.

1. **Preliminary consideration of co-interventions**

a. Are the (pre-specified) co-interventions likely to be administered in the context of this study?

b List additional co-interventions, if any, specific to the setting of this particular study.

1. ***Co-interventions***

In the table below, “critically important” co-interventions are those for which, in the context of this study, adjustment is expected to lead to a clinically important change in the estimated effect of the intervention. “Validity” refers to whether the variables fully measure the co-intervention, while “reliability” refers to the precision of the measurement (more measurement error means less reliability).

| Co-intervention | Is the co- | Did the authors demonstrate that | Is the co-intervention measured | Is presence of this co- |  |
| --- | --- | --- | --- | --- | --- |
|  | intervention | controlling for this co- | validly and reliably? | intervention likely to favour |  |
|  | critically | intervention was unnecessary? |  | outcomes in the experimental or |  |
|  | important?* |  |  | the control group |  |
|  |  |  |  |  |  |
|  | Yes / No |  | Yes / No / No information | Favour experimental / Favour |  |
|  |  |  |  | comparator / No information |  |
|  |  |  |  |  |  |
|  |  |  |  |  |  |
|  | Yes / No |  | Yes / No / No information | Favour experimental / Favour |  |
|  |  |  |  | comparator / No information |  |
|  |  |  |  |  |  |
|  |  |  |  |  |  |
|  | Yes / No |  | Yes / No / No information | Favour experimental / Favour |  |
|  |  |  |  | comparator / No information |  |
|  |  |  |  |  |  |
|  |  |  |  |  |  |

1. **Risk of bias assessment (cohort-type studies)**

| Bias due to | 1.1 Is confounding of the effect of intervention unlikely in this | | Y / PY / PN / N | [Description] |  |  |
| --- | --- | --- | --- | --- | --- | --- |
| confounding | study? | |  |  |  |  |
|  | **If Y or PY to 1.1:** the study can be considered to be at low risk of | |  |  |  |  |
|  | bias due to confounding and no further signalling questions need | |  |  |  |  |
|  | be considered | |  |  |  |  |
|  |  | |  |  |  |  |
|  | **If N or PN to 1.1**: | |  |  |  |  |
|  |  | |  |  |  |  |
|  | 1.2. Were participants analysed according to their initial | | NA / Y / PY / PN / N / NI | [Description] |  |  |
|  | intervention group throughout follow up? | |  |  |  |  |
|  | **If Y or PY to 1.2**, answer questions 1.4 to 1.6, which relate to | |  |  |  |  |
|  | baseline confounding | |  |  |  |  |
|  |  | |  |  |  |  |
|  | 1.3. **If N or PN to 1.2**: Were intervention discontinuations or | | NA / Y / PY / PN / N / NI | [Description] |  |  |
|  | switches unlikely to be related to factors that are prognostic for | |  |  |  |  |
|  | the outcome? | |  |  |  |  |
|  | **If Y or PY to 1.3**, answer questions 1.4 to 1.6, which relate to | |  |  |  |  |
|  | baseline confounding | |  |  |  |  |
|  | **If N or PN to 1.1 and 1.2 and 1.3**, answer questions 1.7 and 1.8, | |  |  |  |  |
|  | which relate to time-varying confounding | |  |  |  |  |
|  |  | |  |  |  |  |
|  | **If Y or PY to 1.2, or Y or PY to 1.3** | |  |  |  |  |
|  |  | |  |  |  |  |
|  | 1.4. Did the authors use an appropriate analysis method | | NA / Y / PY / PN / N / NI | [Description] |  |  |
|  | that adjusted for all the critically important confounding | |  |  |  |  |
|  | domains? | |  |  |  |  |
|  |  | |  |  |  |  |
|  | 1.5. **If Y or PY to 1.4**: Were confounding domains that were | | NA / Y / PY / PN / N / NI | [Description] |  |  |
|  | adjusted for measured validly and reliably by the variables | |  |  |  |  |
|  | available in this study? | |  |  |  |  |
|  |  | |  |  |  |  |
|  | 1.6. Did the authors avoid adjusting for post-intervention | | NA / Y / PY / PN / N / NI | [Description] |  |  |
|  | variables? | |  |  |  |  |
|  |  | |  |  |  |  |
|  | **If N or PN to 1.2 and 1.3** | |  |  |  |  |
|  |  | |  |  |  |  |
|  | | 1.7. Did the authors use an appropriate analysis method | NA / Y / PY / PN / N / NI | [Description] | |  |
|  | | that adjusted for all the critically important confounding |  |  | |  |
|  | | domains and for time-varying confounding? |  |  | |  |
|  | |  |  |  | |  |
|  | | 1.8. **If Y or PY to 1.7**: Were confounding domains that were | NA / Y / PY / PN / N / NI | [Description] | |  |
|  | | adjusted for measured validly and reliably by the variables |  |  | |  |
|  | | available in this study? |  |  | |  |
|  | |  |  |  | |  |
|  | | **Risk of bias judgement** | Low / Moderate / Serious / | [Support for judgement] | |  |
|  | |  | Critical / NI |  | |  |
|  | | Optional: What is the predicted direction of bias due to | Favours experimental / Favours | [Rationale] | |  |
|  | | confounding? | comparator / Unpredictable |  | |  |
| Bias in | | 2.1. Was selection into the study unrelated to intervention or | Y / PY / PN / N / NI | [Description] | |  |
| selection of | | unrelated to outcome? |  |  | |  |
| participants | |  |  |  | |  |
|  |  | 2.2. Do start of follow-up and start of intervention coincide for | Y / PY / PN / N / NI | [Description] | |  |
| into the | | most subjects? |  |  | |  |
| study | |  |  |  | |  |
|  |  |  |  |  | |  |
|  | | 2.3. **If N or PN to 2.1 or 2.2**: Were adjustment techniques used that | NA / Y / PY / PN / N / NI | [Description] | |  |
|  | | are likely to correct for the presence of selection biases? |  |  | |  |
|  | |  |  |  | |  |
|  | | **Risk of bias judgement** | Low / Moderate / Serious / | [Support for judgement] | |  |
|  | |  | Critical / NI |  | |  |
|  | | Optional: What is the predicted direction of bias due to selection of | Favours experimental / Favours | [Rationale] | |  |
|  | | participants into the study? | comparator / Towards null |  | |  |
|  | |  | /Away from null / Unpredictable |  | |  |
| Bias in | | 3.1 Is intervention status well defined? | Y / PY / PN / N / NI | [Description] | |  |
| measurement | | 3.2 Was information on intervention status recorded at the time of | Y / PY / PN / N / NI | [Description] | |  |
| of | | intervention? |  |  | |  |
| interventions | |  |  |  | |  |
|  |  | 3.3 Was information on intervention status unaffected by | Y / PY / PN / N / NI | [Description] | |  |
|  | | knowledge of the outcome or risk of the outcome? |  |  | |  |
|  | | **Risk of bias judgement** | Low / Moderate / Serious / | [Support for judgement] | |  |
|  | |  | Critical / NI |  | |  |
|  | | Optional: What is the predicted direction of bias due to | Favours experimental / Favours | [Rationale] | |  |
|  | | measurement of outcomes or interventions? | comparator / Towards null |  | |  |
|  | |  | /Away from null / Unpredictable |  | |  |
| Bias due to | | 4.1. Were the critical co-interventions balanced across intervention | Y / PY / PN / N / NI | [Description] | |  |
| departures | | groups? |  |  | |  |
| from | | 4.2. Were numbers of switches to other interventions low? | Y / PY / PN / N / NI | [Description] | |  |
| intended | |  |  |  | |  |
|  |  | 4.3. Was implementation failure minor? | Y / PY / PN / N / NI | [Description] | |  |
|  | | 51 |  |  | |  |

| interventions | 4.4. **If N or PN to 4,1, 4.2 or 4.3**: Were adjustment techniques used | NA / Y / PY / PN / N / NI | [Description] |  |
| --- | --- | --- | --- | --- |
|  | that are likely to correct for these issues? |  |  |  |
|  |  |  |  |  |
|  | **Risk of bias judgement** | Low / Moderate / Serious / | [Support for judgement] |  |
|  |  | Critical / NI |  |  |
|  | Optional: What is the predicted direction of bias due to departures | Favours experimental / Favours | [Rationale] |  |
|  | from the intended interventions? | comparator / Towards null |  |  |
|  |  | /Away from null / Unpredictable |  |  |
| Bias due to | 5.1 Are outcome data reasonably complete? | Y / PY / PN / N / NI | [Description] |  |
|  |  |  |  |  |
| missing data | 5.2 Was intervention status reasonably complete for those in whom | Y / PY / PN / N / NI | [Description] |  |
|  | it was sought? |  |  |  |
|  | 5.3 Are data reasonably complete for other variables in the analysis? | Y / PY / PN / N / NI | [Description] |  |
|  | 5.4 **If N or PN to 5.1, 5.2 or 5.3**: Are the proportion of participants | NA / Y / PY / PN / N / NI | [Description] |  |
|  | and reasons for missing data similar across interventions? |  |  |  |
|  |  |  |  |  |
|  | 5.5 **If N or PN to 5.1, 5.2 or 5.3**: Were appropriate statistical | NA / Y / PY / PN / N / NI | [Description] |  |
|  | methods used to account for missing data? |  |  |  |
|  | **Risk of bias judgement** | Low / Moderate / Serious / | [Support for judgement] |  |
|  |  | Critical / NI |  |  |
|  | Optional: What is the predicted direction of bias due to missing | Favours experimental / Favours | [Rationale] |  |
|  | data? | comparator / Towards null |  |  |
|  |  | /Away from null / Unpredictable |  |  |
| Bias in | 6.1 Was the outcome measure objective? | Y / PY / PN / N / NI | [Description] |  |
| measurement |  |  |  |  |
|  | 6.2 Were outcome assessors unaware of the intervention received | Y / PY / PN / N / NI | [Description] |  |
| of outcomes | by study participants? |  |  |  |
|  |  |  |  |  |
|  | 6.3 Were the methods of outcome assessment comparable across | Y / PY / PN / N / NI | [Description] |  |
|  | intervention groups? |  |  |  |
|  |  |  |  |  |
|  | 6.4 Were any systematic errors in measurement of the outcome | Y / PY / PN / N / NI | [Description] |  |
|  | unrelated to intervention received? |  |  |  |
|  |  |  |  |  |
|  | **Risk of bias judgement** | Low / Moderate / Serious / | [Support for judgement] |  |
|  |  | Critical / NI |  |  |
|  | Optional: What is the predicted direction of bias due to | Favours experimental / Favours | [Rationale] |  |
|  | measurement of outcomes? | comparator / Towards null |  |  |
|  |  | /Away from null / Unpredictable |  |  |
| Bias in | Is the reported effect estimate unlikely to be selected, on the basis |  |  |  |
| selection of | of the results, from... |  |  |  |
|  | 52 |  |  |  |
| the reported | 7.1. ... multiple outcome *measurements* within the outcome | Y / PY / PN / N / NI | [Description] |  |
| result | domain? |  |  |  |
|  | 7.2 ... multiple *analyses* of the intervention-outcome relationship? | Y / PY / PN / N / NI | [Description] |  |
|  |  |  |  |  |
|  | 7.3 ... different *subgroups*? | Y / PY / PN / N / NI | [Description] |  |
|  |  |  |  |  |
|  | **Risk of bias judgement** | Low / Moderate / Serious / | [Support for judgement] |  |
|  |  | Critical / NI |  |  |
|  | Optional: What is the predicted direction of bias due to selection of | Favours experimental / Favours | [Rationale] |  |
|  | the reported result? | comparator / Towards null |  |  |
|  |  | /Away from null / Unpredictable |  |  |
| Overall bias | **Risk of bias judgement** | Low / Moderate / Serious / | [Support for judgement] |  |
|  |  | Critical / NI |  |  |
|  | Optional: | Favours experimental / Favours | [Rationale] |  |
|  | What is the overall predicted direction of bias for this outcome? | comparator / Towards null |  |  |
|  |  | /Away from null / Unpredictable |  |  |

1. **Risk of bias assessment (case-control studies).**

| Bias due to | 1.1 Is confounding of the effect of intervention unlikely in this | Y / PY / PN / N | [Description] |  |
| --- | --- | --- | --- | --- |
| confounding | study? |  |  |  |
|  | **If Y or PY to 1.1:** the study can be considered to be at low risk of |  |  |  |
|  | bias due to confounding and no further signalling questions need |  |  |  |
|  | be considered |  |  |  |
|  |  |  |  |  |
|  | **If N or PN to 1.1:** |  |  |  |
|  |  |  |  |  |
|  | 1.4. Did the authors use an appropriate analysis method that | NA / Y / PY / PN / N / NI | [Description] |  |
|  | adjusted for all the critically important confounding domains? |  |  |  |
|  |  |  |  |  |
|  | 1.5. **If Y or PY to 1.4**: Were confounding domains that were | NA / Y / PY / PN / N / NI | [Description] |  |
|  | adjusted for measured validly and reliably by the variables |  |  |  |
|  | available in this study? |  |  |  |
|  |  |  |  |  |
|  | 1.6. Did the authors avoid adjusting for post-intervention | NA / Y / PY / PN / N / NI | [Description] |  |
|  | variables? |  |  |  |
|  |  |  |  |  |
|  | **Risk of bias judgement** | Low / Moderate / Serious / | [Support for judgement] |  |
|  |  | Critical / NI |  |  |
|  | Optional: What is the predicted direction of bias due to | Favours experimental / Favours | [Rationale] |  |
|  | confounding? | comparator / Unpredictable |  |  |
| Bias in | 2.4 Were the controls sampled from the population that gave rise | Y / PY / PN / N / NI | [Description] |  |
| selection of | to the cases, or using another method that avoids selection bias? |  |  |  |
| participants |  |  |  |  |
|  | **Risk of bias judgement** | Low / Moderate / Serious / | [Support for judgement] |  |
| into the |  | Critical / NI |  |  |
| Study |  |  |  |  |
|  | Optional: What is the predicted direction of bias due to selection of | Favours experimental / Favours | [Rationale] |  |
|  |  |  |  |  |
|  | participants into the study? | comparator / Towards null |  |  |
|  |  | /Away from null / Unpredictable |  |  |
| Bias in | 3.1 Is intervention status well defined? | Y / PY / PN / N / NI | [Description] |  |
| measurement | 3.2 Was information on intervention status recorded at the time of | Y / PY / PN / N / NI | [Description] |  |
| Of | intervention? |  |  |  |
| interventions | 3.3 Was information on intervention status unaffected by | Y / PY / PN / N / NI | [Description] |  |
|  | knowledge of the outcome or risk of the outcome? |  |  |  |
|  | **Risk of bias judgement** | Low / Moderate / Serious / | [Support for judgement] |  |
|  |  | Critical / NI |  |  |

|  | Optional: What is the predicted direction of bias due to | Favours experimental / Favours | [Rationale] |
| --- | --- | --- | --- |
|  | measurement of outcomes or interventions? | comparator / Towards null |  |
|  |  | /Away from null / Unpredictable |  |
| Bias due to | 4.1. Were the critical co-interventions balanced across intervention | Y / PY / PN / N / NI | [Description] |
| departures | groups? |  |  |
| from | 4.2. Were numbers of switches to other interventions low? | Y / PY / PN / N / NI | [Description] |
| intended | 4.3. Was implementation failure minor? | Y / PY / PN / N / NI | [Description] |
| interventions | **Risk of bias judgement** | Low / Moderate / Serious / | [Support for judgement] |
|  |  | Critical / NI |  |
|  | Optional: What is the predicted direction of bias due to departures | Favours experimental / Favours | [Rationale] |
|  | from the intended interventions? | comparator / Towards null |  |
|  |  | /Away from null / Unpredictable |  |
| Bias due to | 5.1 Was outcome status reasonably complete for those in whom it | Y / PY / PN / N / NI | [Description] |
| missing data | was sought? |  |  |
|  | 5.2 Were data on intervention status reasonably complete? | Y / PY / PN / N / NI | [Description] |
|  | 5.3 Are data reasonably complete for other variables in the analysis? | Y / PY / PN / N / NI | [Description] |
|  | 5.4 **If N or PN to 5.1, 5.2 or 5.3**: Are the proportion of participants | NA / Y / PY / PN / N / NI | [Description] |
|  | and reasons for missing data similar across cases and controls? |  |  |
|  | 5.5 **If N or PN to 5.1, 5.2 or 5.3**: Were appropriate statistical | NA / Y / PY / PN / N / NI | [Description] |
|  | methods used to account for missing data? |  |  |
|  | **Risk of bias judgement** | Low / Moderate / Serious / | [Support for judgement] |
|  |  | Critical / NI |  |
|  | Optional: What is the predicted direction of bias due to missing | Favours experimental / Favours | [Rationale] |
|  | data? | comparator / Towards null |  |
|  |  | /Away from null / Unpredictable |  |
| Bias in | 6.1 Was the definition of case status (and control status, if | Y / PY / PN / N / NI | [Description] |
| measurement | applicable) based on objective criteria? |  |  |
| of outcomes | 6.2 Was the definition of case status (and control status, if | Y / PY / PN / N / NI | [Description] |
|  | applicable) applied without knowledge of the intervention |  |  |
|  | received? |  |  |
|  | **Risk of bias judgement** | Low / Moderate / Serious / | [Support for judgement] |
|  |  | Critical / NI |  |
|  | Optional: What is the predicted direction of bias due to definitions | Favours experimental / Favours | [Rationale] |
|  | of case and control status? | comparator / Towards null |  |
|  |  | /Away from null / Unpredictable |  |
| Bias in | Is the reported effect estimate unlikely to be selected, on the basis |  |  |
| selection of | of the results, from... |  |  |
| the reported | 7.1. ... multiple *definitions of the intervention*? | Y / PY / PN / N / NI | [Description] |
| result |  |  |  |
|  | 7.2 ... multiple *analyses* of the intervention-outcome relationship? | Y / PY / PN / N / NI | [Description] |
|  | 7.3 ... different *subgroups*? | Y / PY / PN / N / NI | [Description] |
|  | **Risk of bias judgement** | Low / Moderate / Serious / | [Support for judgement] |
|  |  | Critical / NI |  |
|  | Optional: What is the predicted direction of bias due to selection of | Favours experimental / Favours | [Rationale] |
|  | the reported result? | comparator / Towards null |  |
|  |  | /Away from null / Unpredictable |  |
|  |  |  |  |
| Overall bias | **Risk of bias judgement** | Low / Moderate / Serious / | [Support for judgement] |
|  |  | Critical / NI |  |
|  | Optional: What is the overall predicted direction of bias? | Favours experimental / Favours | [Rationale] |
|  |  | comparator / Towards null |  |
|  |  | /Away from null / Unpredictable |  |

© 2014 by the authors. All rights reserved. Reproduced with the permission of the authors.
