# Supplementary material for: Risk of Bias in Systematic Reviews of Non-Randomized Studies of Adverse Cardiovascular Effects of Thiazolidinediones and Cyclooxygenase-2 Inhibitors: Application of a New Cochrane Risk of Bias Tool
Source: PLoS Med. 2016 Apr 5;13(4):e1001987. doi: 10.1371/journal.pmed.1001987 (PMC4821619; doi:10.1371/journal.pmed.1001987)
Supplement: S2 Table — (DOCX) [file pmed.1001987.s002.docx]

| Consensus overall risk of bias ratings by study and corresponding reasons for ranking of Loke et al. [1] component studies **Reference numbers for studies are given in Table 1** | | |
| --- | --- | --- |
| ***Component Study*** | ***Overall RoB judgement*** | ***Comments*** |
| ***Cohort Study Design*** | | |
| *Bilik* | Serious | - missing confounders: smoking, comorbidity, diabetes duration, BMI variables |
| *Brownstein* | Serious | - missing confounders: diabetes duration, smoking, BMI (comorbidities included)  - drug use based on prescriptions (not dispensing) - no censoring, use of departures likely (number of people in monotherapy group was much lower than primary analysis – more switches in rosiglitazone group likely) - crude 6 month intervals, risk may be higher in one intervention vs. other |
| *Graham* | Low |  |
| *Hsiao* | Critical | - unadjusted estimates used, no control of confounding variables (no matching) - monotherapy + dual therapy together, users experiencing immediate events (3 prescriptions) were excluded (risk may be higher among one group sooner than other) - rosiglitazone more common amongst dual usage with other drugs |
| *Juurlink* | Low |  |
| *Margolis* | Serious | - limited comorbidities included as confounders (some proxies)  - prevalent and incident diabetic cases included, study selected those who had survived up to 2002 for prevalent cases  - baseline variables likely measured after diagnosis (not necessarily at start of intervention, clinical variables not significant in model selection) - use identified through prescriptions, not claims  - unclear combination/mono therapy groups |
| *Pantalone* | Critical | - missing confounders: pre-existing CVD events, comorbidity variables (but lab measure proxies used) - incident and prevalent cases included, those entering cohort in 1998 with previous diabetes survived to that point (binary adjustment – new/old, no duration considered) - 75% remained on one drug throughout, but unsure if differential switching occurred (however, this is an ITT analysis) - large % of missing data, imputation may not be sufficient (up to 90% missing in some cases) |
| *Tzoulaki* | Moderate | *we assessed the model 3 estimate, as per Loke’s description (Table 2), however unclear if numerical estimate corresponds with this model (compared with figures in Tzoulaki appendix) - 1/3 missing data in model 3 (assumed missing at random) |
| *Walker* | Low |  |
| *Wertz* | Low |  |
| *Winkelmayer* | Low |  |
| *Ziyadeh* | Moderate | - no controlling for diabetes duration |
| ***Case Control Study Design*** | | |
| *Dormuth* | Low |  |
| *Koro* | Serious | - missing confounding variables for: comorbidities (drug proxies), BMI, smoking  - controls may have been healthier than general population (no MI at any point, more conservative estimate) - RG vs. PG reported results not defined a priori (in text, no further details) |
| *Lipscombe* | Moderate | - unadjusted estimate used (but matched on key variables, adjusted vs unadjusted estimates change minimally for other comparisons) |
| *Stockl* | Moderate | - did not control for duration of diabetes, smoking, BMI (but many proxies included) |

1. Loke YK, Kwok CS, Singh S. Comparative cardiovascular effects of thiazolidinediones: systematic review and meta-analysis of observational studies2011 2011-03-17 23:34:35.
