# Supplementary material for: Risk of Bias in Systematic Reviews of Non-Randomized Studies of Adverse Cardiovascular Effects of Thiazolidinediones and Cyclooxygenase-2 Inhibitors: Application of a New Cochrane Risk of Bias Tool
Source: PLoS Med. 2016 Apr 5;13(4):e1001987. doi: 10.1371/journal.pmed.1001987 (PMC4821619; doi:10.1371/journal.pmed.1001987)
Supplement: S3 Table — (DOCX) [file pmed.1001987.s003.docx]

| Consensus overall risk of bias ratings by study and corresponding reasons for ranking of McGettigan and Henry [1] component studies **Reference numbers for the studies are provided in Table 2** | | |
| --- | --- | --- |
| ***Component study*** | ***Overall RoB Judgements*** | ***Comments*** |
| ***Cohort Study Design*** | | |
| *Curtis* | Serious | - Excluded recurrent MI (people dying after recurrent MI are not captured, may be more probable in aspirin only group),  - Drug info only available at discharge (can change over time) - Unsure about degree of missing data (table 1 reports only %, no mention of missing n) |
| *Gislason* | Low | - Case crossover analysis also done to assess significance of missing confounders |
| *MacDonald* | Moderate | - No OTC drugs included (but assessment done to test importance), behavioural confounding variables also missing |
| *Mamdani* | Moderate | - missing confounders: OTC, behavioural variables, drug proxies for comorbidities |
| *Ray, 2002a* | Moderate | - missing confounders: OTC, behavioural variables |
| *Ray, 2002b* | Moderate | - missing confounders: OTC, behavioural variables |
| ***Case Control Study Design*** | | |
| *Bak* | Serious | - Limited direct exposure measures (e.g. prescription drugs as proxies for comorbidities), no behavioural variables included as confounders |
| *Fischer* | Moderate | - missing OTC drug use (likely confounder) - missing data in BMI, smoking |
| *Garcia Rodriquez, 2000* | Moderate | - missing OTC drug use (likely confounder) - missing data in smoking (unsure if differential) |
| *Garcia Rodriquez, 2004* | Moderate | - missing OTC drug use (likely confounder) - can't determine degree of missing data across intervention groups (unlikely to be differential) |
| *Graham* | Low | - Did additional survey to look at unmeasured confounders |
| *Hippisley-Cox* | Moderate | - missing OTC drug use (likely confounder) - analysis using complete data shows difference for naproxen |
| *Johnsen* | Moderate | - missing confounders: OTC, behavioural variables |
| *Kimmel, 2004* | Serious | - missing OTC drug use (likely confounder) - Population-based controls (healthier, may have poor recall of NSAID use) - recall bias (exposure measured retrospectively by interviewing) - high % missing data (unlikely to be differential but may misclassify some MI events) - low participation rate |
| *Kimmel, 2005* | Serious | Same as Kimmel, 2004 |
| *Levesque* | Moderate | - missing confounders: OTC, behavioural variables |
| *McGettigan* | Moderate | - Recall bias possible (exposure measured retrospectively through interviews) - possible info about MI risk circulating 2003-2004, recall may be different (3.3) |
| *Schlienger* | Moderate | - missing confounders: OTC, behavioural variables - 20-30% missing data (unlikely to be differential) |
| *Solomon, 2002* | Serious | - missing confounders: OTC, behavioural variables - Controls were AMI-free for whole study period (healthier than average population) - did not discuss intervention switching within 180 days prior to event  - Many analyses conducted (no reasons given for selecting 180 days vs. a year, as exposure period, etc.) |
| *Solomon, 2004* | Moderate | - Compared with Medicare survey of non-database variables (population based) - controls may be healthier than average population (no AMI throughout study period) |
| *Watson* | Moderate | - Populations are very similar - all have RA (unlikely to have significant confounding due to OTC, BMI – although these variables are not included) - missing data, unclear if differential |

1. McGettigan P, Henry D. Cardiovascular risk and inhibition of cyclooxygenase: A systematic review of the observational studies of selective and nonselective inhibitors of cyclooxygenase 2. JAMA. 2006;296(13):1633-44. doi: 10.1001/jama.296.13.jrv60011.
